# Variational Disentanglement for Rare Event Modeling

**Published:** 2020-09-17

**Authors:** Zidi Xiu, Chenyang Tao, Michael Gao, Connor Davis, Benjamin Goldstein, Ricardo Henao

**Affiliations:** Duke University

## Abstract

Combining the increasing availability and abundance of healthcare data and the current advances in machine learning methods have created renewed opportunities to improve clinical decision support systems. However, in healthcare risk prediction applications, the proportion of cases with the condition (label) of interest is often very low relative to the available sample size. Though very prevalent in healthcare, such imbalanced classification settings are also common and challenging in many other scenarios. So motivated, we propose a variational disentanglement approach to semi-parametrically learn from rare events in heavily imbalanced classification problems. Specifically, we leverage the imposed extreme-distribution behavior on a latent space to extract information from low-prevalence events, and develop a robust prediction arm that joins the merits of the generalized additive model and isotonic neural nets. Results on synthetic studies and diverse real-world datasets, including mortality prediction on a COVID-19 cohort, demonstrate that the proposed approach outperforms existing alternatives.

## INTRODUCTION

Early identification of in-hospital patients who are at imminent risk of life-threatening events, *e.g*., death, ventilation or intensive care unit (ICU) transfer, is a critical subject in clinical care (Bedoya et al. 2019). Especially during a pandemic like COVID-19, the needs for healthcare change dramatically. With the ability to accurately predict the risk, an automated triage system will be well-positioned to help clinicians better allocate resources and attention to those patients whose adverse outcomes can be averted if early intervention efforts were in place.

Despite the great promise it holds, with the richness of modern Electronic Health Record (EHR) repositories, the construction of such a system faces practical challenges. A major obstacle is the scarcity of patients experiencing adverse outcomes of interest. In the COVID-19 scenario, which we consider in our experiments, the mortality of patients tested positive at the Duke University Health System (DUHS) is slightly lower than 3%. Further, in another typical EHR dataset we consider, less than 5% of patients are reported to suffer adverse outcomes (ICU transfer or death). In these *low-prevalence* scenarios, commonly seen in clinical practice, standard classification models such as logistic regression suffer from *majority domination*, in which models tend to favor the prediction accuracy of majority groups. This is clearly undesirable for critical-care applications, given the high false negative rates (Type-II error), in which patients in urgent need of care could be falsely categorized.

Situations where the distribution of labels is highly skewed and the accuracy of the minority class bears particular significance (Dal Pozzolo et al. 2017; Lu, Guo, and Li 2020; Machado and Lopes 2020) have been associated with the name *imbalanced dataset* (He and Garcia 2009), whereas the methods dealing with such cases are coined *extreme classification* (Zong, Huang, and Chen 2013). Under such a setting, the lack of representation of minority cases severely undermines the ability of a standard learner to discriminate, relative to balanced datasets (Mitchell 1999). Consequently, these solutions do not generalize well on minority classes, where the primary interest is usually focused.

To address such a dilemma, several remedies have been proposed to account for the imbalance between class representations. One of the most popular strategies is the sampling-based adjustment, where during training, a model oversamples the minority classes (or undersamples the majority classes) to create balance artificially (Drummond, Holte et al. 2003). To overcome the biases and the lack of information that naive sampling adjustments might induce, variants have been proposed to maximally preserve the clustering structure of the original dataset (Mani and Zhang 2003; Yen and Lee 2009) and to promote diversity of oversampling schemes (Han, Wang, and Mao 2005). Alternatively, cost-sensitive weighting where minority losses are assigned larger weights provides another popular option via tuning the relative importance of minority classes (Elkan 2001; Munro et al. 1996; Zhou and Liu 2005).

While the above two strategies introduce heuristics to alleviate the issues caused by class imbalance, importance sampling (IS) offers a principled treatment that flexibly combines the merits of the two (Hahn and Jeruchim 1987; Heidelberger 1995). Each example is sampled with the probability of a pre-specified importance weight, and with the weight’s inverse when accounting for the relative contribution in the overall loss. This helps to flexibly tune the representation of rare events during training, without biasing the data distribution (Heidelberger 1995; Shimodaira 2000; Gretton et al. 2009). It is important to note that, poor choice of importance weights may result in uncontrolled variance that destabilizes training (Robert and Casella 2013; Botev and Kroese 2008), calling for adaptive (Rubinstein and Kroese 2013) or variance reduction schemes (Rubinstein and Kroese 2016) to protect against such degeneracy.

Apart from the above strategies that fall within the standard empirical risk minimization framework, recent developments explicitly seek better generalization for the minority classes. One such example is the *one-class classification* that aims to capture one target class from a general population (Tax 2002). *Meta-learning* and *few-shot learning* strategies instead trying to transfer the knowledge learned from data-rich classes to facilitate the learning of data-scarce classes (Böhning, Mylona, and Kimber 2015; Finn, Abbeel, and Levine 2017). Additionally, non-cross-entropy based losses or penalties have been proved useful to imbalanced classification tasks (Weinberger and Saul 2009; Huang et al. 2016). For instance, the Focal loss (Lin et al. 2017) up-weights the harder examples, and Cao et al. (2019) introduced a label-distribution-aware margin loss encouraging minority classes having larger margins.

In this work, we present a novel solution called *variational inference for extremals* (VIE), capitalizing on the learning of more generalizable representations for the minority classes. Our proposal is motivated by the observation that the statistical features of “rarity” have been largely overlooked in the current literature of rare-event modeling. And the uncertainties of rare-events are often not considered. Framed under the Variational Inference framework, we formulate our model with the assumption that the extreme presentation of (unobserved) latent variables can lead to the occurrence (or the inhibition) of rare events. This encourages the accurate characterization of the tail distribution of the data representation, which has been missed by prior work to the best of our knowledge. Building upon the state-of-the-art machine learning techniques, our solution features the following contributions: (*i*) the model accounts for representation uncertainty based on variational inference; (*ii*) the adoption of mixed Generalized Pareto priors to promote the learning of heavy-tailed feature representations; and (*iii*) integration of additive isotonic regression to disentangle representation and facilitate generalization. We demonstrate how our framework facilitates both model generalization and interpretation, with strong empirical performance reported across a wide-range of benchmarks.

## BACKGROUND

To simplify our presentation, we focus on the problem of rare event classification for binary outcomes. The generalization to the multiple-class scenario is simple and presented in the Supplementary Material (SM)^1^. Let $D={\left\{x_{i},y_{i}\right\}}_{i=1}^{N}$ be a dataset of interest, where *x*_*i*_ and *y*_*i*_ denote predictors and outcomes, respectively, and *N* is the sample size. Without loss of generality, we denote *y* = 1 as the minority event label (indicating the occurrence of an event of interest), and *y* = 0 as the majority label.

In the following, we will briefly review the three main techniques we used in this work, namely, *variational inference* (VI), *extreme value theory* (EVT), and *additive isotonic regression*. VI allows for approximate maximum likelihood inference while accounting for data uncertainty. EVT provides a principled and efficient way to model extreme, heavy-tailed representations. Additive isotonic regression further introduces monotonic constraints to *disentangle* the contribution of each latent dimension to the outcome.

### Variational inference

Consider a latent variable model *p*_*θ*_(*v*, *z*) = *p*_*θ*_(*v*|*z*)*p*(*z*), where $v\in {\mathbb{R}}^{m}$ is the observable data, $z\in {\mathbb{R}}^{p}$ is the unobservable latent variable, and *θ* represents the parameters of the likelihood model, *p*_*θ*_(*v*|*z*). The marginal likelihood *p*_*θ*_(*v*) = ∫ *p*_*θ*_(*v*, *z*)d*z* requires integrating out the latent *z*, which typically, for complex distributions, does not enjoy a closed-form expression. This intractability prevents direct maximum likelihood estimation for *θ* in the latent variable setup. To overcome this difficulty, Variational Inference (VI) optimizes computationally tractable variational bounds to the marginal log-likelihood (Kingma and Welling 2014; Chen et al. 2018). Concretely, the most popular choice of VI optimizes the following Evidence Lower Bound (ELBO):

(1) $$\text{ELBO}\left(v;p_{\theta }(v,z),q_{\phi }(z|v)\right)≜E_{Z\sim q_{\phi }(z|v)}\left[\text{log }\frac{p_{\theta }(v,Z)}{q_{\phi }(Z|v)}\right]***\leq \text{log }p_{\theta }(v),$$

where *q*_*φ*_(*z*|*v*) is an approximation to the true (unknown) posterior *p*_*θ*_(*z*|*v*), and the inequality is a direct result of Jensen’s inequality. The variational gap between the ELBO and true marginal log-likelihood, *i.e*., log*p*_*θ*_(*v*) − ELBO(*v*; *p*_*θ*_(*v*, *z*), *q*_*φ*_(*z*|*v*)), is given by the Kullback–Leibler (KL) divergence between posteriors, *i.e*., $\text{KL}\left(q_{\phi }(z|v)\|p_{\theta }(z|v)\right)=E_{Z\sim q_{\phi }(z|v)}\left[\text{log }q_{\phi }(Z|v)\right]-E_{Z\sim q_{\phi }(z|v)}\left[\text{log }p_{\theta }(Z|v)\right]$, which implies that the ELBO tightens as *q*_*φ*_(*z*|*v*) approaching the true posterior *p*_*θ*_(*z*|*v*). For estimation, we seek parameters *θ* and *φ* that maximize the ELBO in (1).

Given a set of observations ${\left\{v_{i}\right\}}_{i=1}^{N}$ sampled from data distribution *v* ~ *p*_*d*_(*v*), maximizing the expected ELBO is also equivalent to minimizing the KL divergence KL(*p*_*d*_(*v*) ∥ *p*_*θ*_(*v*)) between the empirical and model distributions. When *p*_*θ*_(*v*|*z*) and *q*_*φ*_(*z*|*v*) are specified as neural networks, the resulting architecture is commonly known as the *variational auto-encoder* (VAE) (Kingma and Welling 2014), where *q*_*φ*_(*z*|*v*) and *p*_*θ*_(*v*|*z*) and are known as *encoder* and *decoder*, respectively. Note that *q*_*φ*_(*z*|*v*) is often used for subsequent inference tasks on new data.

### Extreme Value Theory

Extreme Value Theory (EVT) provides a principled probabilistic framework for describing events with extremely low probabilities, which we seek to exploit for better rare event modeling. In particular, we focus on the *exceedance* models, where we aim to capture the asymptotic statistical behavior of values surpassing an extreme threshold (Davison and Smith 1990; Tao et al. 2017), which we briefly review below following the notation of Coles et al. (2001). Without loss of generality, we consider exceedance to the right, *i.e*., values greater than a threshold *u*. For a random variable *X*, the conditional cumulative distribution of exceedance level *x* beyond *u* is given by *F*_*u*_(*x*) = *P*(*X* − *u* ≤ *x*|*X* > *u*) = $\frac{F(x+u)-F(u)}{1-F(u)}$, where *x* > 0 and *F*(*x*) denotes the cumulative density function for *X*.

A major result from EVT is that under some mild regularity conditions, *e.g*., continuity at the right end of *F*(*x*) and others, *F*_*u*_(*x*) will converge to the family of Generalized Pareto Distributions (GPD) regardless of *F*(*x*), as *u* approaches the right support boundary of *F*(*x*) (Balkema and De Haan 1974; Pickands III et al. 1975), *i.e*., ${\text{lim}}_{u\to \infty }F_{u}(x)\overset{L_{\infty }}{\to }G_{\xi ,\sigma ,u}(x)$ (Falk, Hüsler, and Reiss 2010), where GPD_*ξ,σ,u*_(*x*) is of the form

(2) $$G_{\xi ,\sigma ,u}(x)=\{\begin{array}{ll} 1-{\left[1+\xi (x-u)/\sigma \right]}^{-\frac{1}{\xi }}, & \text{ if }\xi \neq 0\\ 1-\text{exp}[-(x-u)/\sigma ], & \text{ if }\xi =0 \end{array}$$

where *σ* is a positive scale parameter. When *ξ* < 0 the exceedance *x* has bounded support 0 ≤ *x* ≤ *u* − *σ/ξ*, otherwise when *ξ* ≥ 0, *x* is unbounded. A major implication of this asymptotic behavior is that, for modeling extreme values, one only needs to fit extreme samples to the log-likelihood function of the GPD.

### Additive Isotonic Regression

Also known as monotonic regression, isotonic regression is a non-parametric regression model that constrains the relation between predictor and outcome to be monotonic, (*e.g*., non-decreasing *f*(*a*) ≤ *f*(*b*) for *a* ≤ *b*) (Barlow et al. 1972). Such monotonic constraint is a natural and flexible extension to the standard linear relation assumed by many statistical models. To accommodate multi-covariate predictors, additive isotonic regression combines isotonic models for each individual one-dimensional predictor (Bacchetti 1989). Standard implementations often involve specialized algorithms, such as local scoring algorithms (Hastie 2017) and the alternating conditional expectation (ACE) method of Breiman and Friedman (1985). All these approaches typically require costly iterative computations and are not scalable to large datasets. Here we consider recent advances in unconstrained monotonic neural networks, which allow for efficient and flexible end-to-end learning of monotonic relations with robust neural nets based on standard training schemes such as stochastic gradient descent (Sill 1998; Wehenkel and Louppe 2019).

## VARIATIONAL INFERENCE OF EXTREMALS

The proposed model is based on the hypothesis that *extreme events are driven by the extreme values of some latent factors*. Specifically, we propose to recast the learning of low-prevalence events into the learning of extreme latent representations, thus amortizing the difficulties associated with directly modeling rare events as outcomes. To allow for more efficient learning from the rare events, we make some further assumptions to regularize the latent representation: (*i*) *effect disentanglement*: the contribution from each dimension of the latent representation to the event occurrence is additive; (*ii*) *effect monotonicity*: there is a monotonic relation between the outcome likelihood and the values of each dimension of the latent representation. The key to the proposed approach is using an additive isotonic neural network to model the one-dimensional disentangled monotonic relations from a latent representation, which is obtained via variational inference. Specifically, we impose an EVT prior to explicitly capture the information from the few minority group samples into the tail behavior of the extreme representation. Below we provide the rationale for our choices followed by a description of all model components.

### Disentanglement & additive isotonic regression.

Consistent with assumptions (*i*) and (*ii*), we posit a scenario in which the underlying representation of extreme events is more frequent at the far end of the representation spectrum, for which additive isotonic regression is ideal. The disentanglement consists of modeling each latent dimension individually, thus avoiding the curse of dimensionality when modeling combinatorial effects with few examples. Further, the monotonicity constraint imposed by the isotonic regression model restricts possible effect relations, thereby improving generalization error by learning with a smaller, yet still sufficiently expressive, class of models (Bacchetti 1989).

### EVT & VI.

Note that the spread of representation of extreme events is expected to be more uncertain relative to those of the normal, more abundant events, due to a few plausible causes: (*i*) extreme events represent the breakdown of system normality and are expected to behave in uncertain ways; (*ii*) there is only a small number of examples available for the extreme events, so the learned feature encoder will tend to be unreliable. As a result, it is safely expected that the encoded features associated with the extremes events will lie outside the effective support of the Gaussian distribution assumed by the standard VI model. In other words, the representation of the events can manifest as a heavy-tailed distribution. This will compromise the validity and generalizability of a prediction model if not dealt with appropriately. So motivated, we explicitly model the distribution of the extreme underlying representations via EVT. Using EVT, we decouple the learning of the tail end of the representation distribution. Since EVT-based estimation only requires very few parameters, it allows for accurate modeling with a small set of tail-end samples. Further, in combination with the variational inference framework, it accounts for representation uncertainty via the use of a stochastic encoder, which further strengthens model robustness.

### Benefits of heavy-tailed modeling.

**Figure 1:**
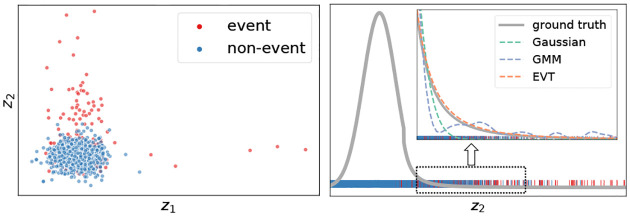
Left: Distribution of a two-dimensional latent space *z* where the long tail associates with higher risk. Right: Tail estimations with different schemes for the long-tailed data in one-dimensional space. EVT provides more accurate characterization comparing to other mechanisms.

A few other considerations further justify modeling with a heavy-tailed distribution for the extreme event representation. One obvious benefit is that it allows better model resolution along the representation axis, *i.e*., better risk stratification. For light-tail representations, extreme examples are clustered in a narrow region where the tail vanishes, thus a standard (light-tailed) learning model will report the average risk in that region. However, if the representations are more spread out, then there is a more gradual change in risk, which can be better captured, as shown in Figure 1. Another argument for favoring heavy-tailed representations is that heavy-tailed phenomena are very common in nature (Bryson 1974), and these tail samples are often encoded less robustly due to the lack of training examples. Allowing long-tail representations relieves the burden of an encoder.

### Model structure.

We consider latent variable model *p*_*θ*_(*y*, *x*, *z*) = *p*_*θ*_(*y*|*z*)*p*_*θ*_(*x*|*z*)*p*(*z*), where *v* = {*x*, *y*} are the observed variables. Under the VI framework, similar to (1) we write the ELBO(*v*; *p*_*θ*_(*v*, *z*), *q*_*φ*_(*z*|*v*)) as

(3) $$E_{Z\sim q_{\phi }(z|v)}\left[\text{log }p_{\theta }(y|Z)\right]+E_{Z\sim q_{\phi }(z|v)}\left[\text{log }p_{\theta }(x|Z)\right]-\text{KL}\left(q_{\phi }(z|v)\|p(z)\right)$$

where *p*_*θ*_(*y*|*z*) is specified as an additive isotonic regression model, *p*(*z*) is modeled with EVT, and the approximate posterior, *q*_*φ*_(*z*|*v*), is specified as an inverse auto-regressive flow. Note that unlike in the standard ELBO in (3), we have dropped the term $E_{Z\sim q_{\phi }(z|v)}\left[\text{log }p_{\theta }(x|z)\right]$ because we are not interested in modeling the covariates. Note this coincides with the *variational information bottleneck* (VIB) formulation (Alemi et al. 2016). Additionally, the posterior *q*_*φ*_(*z*|*v*) will not be conditioned on *y*, but only on *x*, because in practice, the labels *y* are not available at inference time. Specifically, we rewrite the objective in (3) as

(4) $$\Psi_{\beta }\left(x,y;p_{\theta }(y|z),q_{\phi }(z|x)\right)=E_{Z\sim q_{\phi }(z|x)}\left[\text{log }p_{\theta }(y|Z)\right]-\beta \text{KL}\left(q_{\phi }(z|x)\|p(z)\right),$$

where *β* is a hyperparameter controlling the relative contribution of the KL term to the objective. Below we provide details for each component of the proposed approach.

### Decoder: Additive Monotonic Neural Network

First, let us consider the following monotone mapping $\int_{l}^{z}h(s;\theta )ds+\gamma$, consisting on integrating a non-negative function *h*(*s*; *θ*) specified as a neural network with one-dimensional input, *s*, and parameterized by *θ*. The choice of the lower end *l* is arbitrary, and *γ* is a bias term. For multi-dimensional latent representation $z\in {\mathbb{R}}^{p}$, we write the additive monotonic neural network (AMNN) as

(5) $$H(z;\theta )=\overset{p}{\underset{j}{\sum }}\left[\alpha_{j}\int_{l}^{z_{j}}h_{j}(s;\theta )ds\right]+\gamma ,$$

where *α*_*j*_ serves as a weight which controlling the effect directions. In other words, when *α*_*j*_ > 0, it can be interpreted as an event stimulator; otherwise it is an event blocker. To ensure *h*(*s*; *θ*) is non-negative, we apply exponential activation function to the network’s output. The integration of *z* is conducted with numerical integration by the RiemannStieltjes method (Davis and Rabinowitz 2007).

From (5) we obtain log *p*_*θ*_(*y*|*z*) = *ℓ*_CLL_(*y*, *H*(*z*; *θ*)), where $\ell_{\text{CLL}}(y,a)=\text{log}\{1_{y=1}(y)(1-\text{exp}(-\text{exp}(a)))+1_{y=0}(y)\text{exp}(-\text{exp}(a))\}$ is the complementary log-log (CLL) link, where $1_{\left(\cdot \right)}$ is the indicator function. We prefer CLL over the standard logistic link since the CLL link is more sensitive at the tail end (Aranda-Ordaz 1981).

### Latent Prior: Gaussian GPD Mixture

To better capture the tail behavior of the latent representation, we assume random variable *Z* ~ *p*(*z*) is a mixture of a standard Gaussian distribution truncated at *u* and a GPD for modeling the tail end thresholded at *u*, *i.e*., *F*(*z*) = Φ(*z*) when *z* ≤ *u* and *F*(*z*) = Φ(*u*) + (1 − Φ(*u*))*G*_*ξ,σ*_(*z* − *u*) when *z* > *u*, where Φ(*z*) denotes the CDF of a standard Gaussian distribution. Note that for *z* > *u*, *F*(*z*) can be expressed as a GPD with parameters ($\tilde{\xi }$, $\tilde{\sigma }$, $\tilde{u}$) (McNeil 1997), where $\tilde{\xi }=\xi$ and if *ξ* ≠ 0, $\tilde{\sigma }=\sigma {\left(1-\Phi (u)\right)}^{\xi }$ and $\tilde{u}=u-\tilde{\sigma }\left({\left(1-\Phi (u)\right)}^{-\xi }-1\right)/\xi$. Otherwise, when *ξ* = 0, $\tilde{\sigma }=\sigma$ and $\tilde{u}=u+\tilde{\sigma }\text{log}(1-\Phi (u))$. Consequently, the CDF for the mixed GPD is given by

(6) $$F(z)=1_{\left(-\infty ,u\right]}(z)\Phi (z)+1_{\left(u,\infty \right)}(z)G_{\tilde{u},\xi ,\tilde{\sigma }}(z).$$

For simplicity, we denote the set of parameters in GPD as *ψ*={*ξ*_GPD_, *σ*_GPD_} and the threshold *u* is a user-defined parameter. In the experiments we set *u* to Φ^−1^(0.99).

### Latent Posterior: Inverse Autoregressive Flow

Considering we have adopted a long-tailed GPD prior, we seek a posterior approximation *q*_*φ*_(*z*|*x*) that is: (*i*) a flexible parameterization to approximate arbitrary distributions; and (*ii*) with a tractable likelihood to be able to evaluate the KL(*q*_*φ*_(*z*|*x*) ∥ *p*(*z*)) exactly. We need (*i*) because the true posterior is likely to exhibit heavy-tailed behavior due to the extended coverage of the GPD prior, and (*ii*) is to ensure accurate and low-variance Monte Carlo estimation of the KL-divergence at the tail end of the prior. These requirements invalidate some popular choices, *e.g*., a standard Gaussian posterior is light-tailed, and the implicit neural-sampler-based posterior typical in the work of adversarial variational Bayes (Mescheder, Nowozin, and Geiger 2017), does not have a tractable likelihood.

One model family satisfying the above two requirements is known as the generative flows (Rezende and Mohamed 2015), where simple invertible transformations with tractable log Jacobian determinants are stacked together, transforming a simple base distribution into a complex one, while still having closed-form expressions for the likelihood. In this work, we consider the *inverse autoregressive flow* (IAF) model (Kingma et al. 2016). The flow chain is built as:

(7) $$z_{t}=\mu_{t}+\sigma_{t}⊙z_{t-1},\text{ for }1\leq t\leq T,$$

where $\mu_{t}\in {\mathbb{R}}^{p}$ and $\sigma_{t}\in {\mathbb{R}}^{p}$ are learnable parameters, ⊙ denotes the element-wise product, *z*_0_ is typically drawn from a *p*-dimensional Gaussian distribution, *z*_0_ ~ 𝒩(*μ*_0_, $\text{Diag}\left(\sigma_{0}^{2}\right)$) where *μ*_0_ and *σ*_0_ are obtained from an initial encoder defined by a neural network given input *x* with parameter *φ*. A sample from the posterior *q*_*φ*_(*z*|*x*) is given by *z*_*T*_, obtained by “flowing” *z*_0_ through (7). Provided the Jacobians $\frac{d\mu_{t}}{dz_{t-1}}$ and $\frac{d\sigma_{t}}{dz_{t-1}}$ are strictly upper triangular (Papamakarios, Pavlakou, and Murray 2017), we obtain the following closed-form expression for the log posterior

(8) $$\text{log }q(z|x)=\text{log }q\left(z_{0}|x\right)-\sum_{t=1}^{T}\text{log }\text{det}\left|\frac{dz_{t}}{dz_{t-1}}\right|***=-\sum_{j=1}^{p}\left(\frac{1}{2}e_{j}^{2}+\frac{1}{2}\text{log}(2\pi )+\sum_{t=0}^{T}\text{log }\sigma_{t,j}\right)\text{,}$$

where *e*_*j*_ = (*x*_*j*_ − *μ*_0*,j*_)*/σ*_0*,j*_ for the *j*th dimension.

### Posterior Match with Fenchel Mini-Max Learning

We consider an additional modification that explicitly encourages the match of the aggregated posterior *q*_*φ*_(*z*) = ∫ *q*_*φ*_(*z*|*x*)*p*_*d*_(*x*)d*x* to the prior *p*(*z*), which has been reported to be vastly successful at improving VAE learning (Mescheder, Nowozin, and Geiger 2017). In our case, *q*_*φ*_(*z*) does not have a closed-form expression for the likelihood ratio of the KL formulation, which motivates us to use a sample-based estimator. We consider the mini-max KL estimator based on the Fenchel duality (Tao et al. 2019; Dai et al. 2018). Concretely, recall the KL can be expressed in its Fenchel dual form^2^

(9) $$\Gamma \left(p,q_{\phi },\nu \right)=E_{Z\sim q_{\phi }(z)}[\nu (Z)]-E_{Z^{\prime }\sim p(z)}\left[\text{exp}\left(\nu \left(Z^{\prime }\right)\right)\right]***\text{KL}\left(q_{\phi }(z)\|p(z)\right)=\underset{\nu \in ℱ}{\text{max}}\;\Gamma (p,q,\nu ),$$

where *ν*(*z*) is commonly known as the critic function in the adversarial learning literature, and we maximize wrt *ν*(*z*) in the space of all functions ℱ, modeled with a deep neural network. We use (4) and (9) to derive an augmented ELBO that further penalizes the discrepancy between the aggregated posterior and the prior, *i.e*., Ψ_*β*_(*x*, *y*; *p*_*θ*_(*y*|*z*), *q*_*φ*_(*z*|*x*)) − *λ*KL(*q*_*φ*_(*z*) ∥ *p*(*z*)), where *λ* is a regularization hyperparameter (Chen, Feng, and Lu 2018). Solving for this objective results in the following mini-max game

(10) $$\underset{\theta ,\phi }{\text{max}}\;\underset{\nu }{\text{min}}\;\Psi_{\beta }\left(x,y;p_{\theta }(y|z),q_{\phi }(z|x)\right)-\lambda \Gamma \left(p_{\theta },q_{\phi },\nu \right),$$

where *β* and *λ* are regularization hyperparameters. In a similar vein to *β*-VAE and adversarial variational Bayes (AVB), our objective leverages *β*, *λ* > 0 to balance the prediction accuracy and the complexity of the latent representation via KL regularization. Further, from Ψ_*β*_(*x*, *y*; *p*_*θ*_(*y*|*z*), *q*_*φ*_(*z*, *x*)) in (4), note that the decoder *p*_*θ*_(*y*|*z*) is obtained from the additive neural network in (5), *p*_*ψ*_(*z*) is the Gaussian GPD mixture with CDF in (6), *q*_*φ*_(*z*|*x*) is the autoregressive flow implied by (7) and *ν*(*z*; *ω*) is the critic function specified as a neural network and parameterized by *ω*.


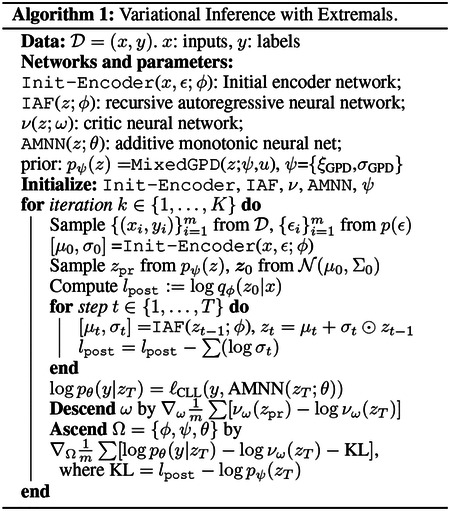


To avoid collapsing to suboptimal local minima, we train the encoder arm more frequently to compensate for the detrimental posterior lagging phenomenon (He et al. 2019). The pseudo-code for the proposed VIE is summarized in Algorithm 1 and detailed architecture can be found in the SM.

## RELATED WORK

### Rare-event modeling with regression.

Initiated by King and Zeng (2001), the discussion on how to handle the unique challenges presented by rare-event data for regression models has attracted extensive research attention. The statistical literature has mainly focused on bias correction for sampling (Fithian and Hastie 2014) and estimation (Firth 1993), driven by theoretical considerations in maximum likelihood estimation. However, their assumptions are often violated in the face of modern datasets (Sur and Candès 2019), characterized by high-dimensionality and complex interactions. Our proposal approaches a solution from a representation learning perspective (Bengio, Courville, and Vincent 2013), by explicitly exploiting the statistical regularities of extreme values to better capture extreme representations associated with rare events.

### Re-sampling and loss correction.

Applying statistical adjustments during model training is a straightforward solution to re-establish balance, but often associated with obvious caveats. For example, the popular down-sampling and up-sampling (He and Garcia 2009) discard useful information or introduce artificial bias, exacerbating the chances of capturing spurious features that may harm generalization (Drummond, Holte et al. 2003; Cao et al. 2019), and their performance gains may be limited (Byrd and Lipton 2019). While traditionally tuned by trial and error, recent works have explored automated weight adjustments (Lin et al. 2017; Zhang et al. 2020), and principled loss correction that factored in class-size differences (Cui et al. 2019). Our contribution is orthogonal to these developments and promises additional gains when used in synergy.

### Transferring knowledge from the majority classes.

Adapting the knowledge learned from data-rich classes to their under-represented counterparts has shown success in few-shot learning, especially in the visual recognition field (Wang, Ramanan, and Hebert 2017; Chen et al. 2020), and also in the clinical setting (Böhning, Mylona, and Kimber 2015). However, their success often critically depends on strong assumptions, the violation of which typically severely undermines performance (Wang et al. 2020). Related are the one-class classification (OCC) models (Tax 2002), assuming stable patterns for the majority over the minority classes. Our assumptions are weaker than those made in these model categories, and empirical results also suggest the proposed VIE works more favorably in practice (see experiments).

## EXPERIMENTS

We carefully evaluate the proposed VIE on a diverse set of realistic synthetic data and real-world datasets with different degrees of imbalance. Our implementation is based on PyTorch, and code to replicate our experiments are available from https://github.com/ZidiXiu/VIE/. We provide additional experiments and analyses in the SM.

### Baseline Models

We consider the following set of competing baselines to compare the proposed solution: LASSO regression (Tibshirani 1996), MLP with re-sampling and re-weighting (MLP), Importance-Weighting model (IW) (Byrd and Lipton 2019), FOCAL loss (Lin et al. 2017), Label-Distribution-Aware Margin loss (LDAM) (Cao et al. 2019), and SVD based one-class classification model (Deep-SVDD) (Ruff et al. 2018). We tune the hyper-parameters of baseline models on the validation dataset, and pick best performing hyper-parameters to evaluate test set performance. For detailed settings please refer to the SM.

### Evaluation Metrics

To quantify model performance, we consider AUC and AUPRC. AUC is the area under the Receiver Operating Characteristic (ROC) curve, which provides a threshold-free evaluation metric for classification model performance. AUC summarizes the trade-off between True Positive Rate (TPR) and False Positive Rate (FPR). AUPRC summarizes the trade-off between TPR and True Predictive Rate. Specifically, it evaluates the area under Precision-Recall (PR) curve. We discuss other metrics in the SM. In simulation studies, we repeat simulation ten times to obtain empirical AUC and AUPRC confidence intervals. For real world datasets, we applied bootstrapping to estimate the confidence intervals.

### Ablation study for VIE

VIE applies a few state-of-art techniques in variational inference in order to achieve optimal performance. In this section, we decouple their contributions via an ablation study, to justify the necessity of including those techniques in our final model. To this end, we synthesize a semi-synthetic dataset based on the Framingham study (Mitchell et al. 2010), a long-term cardiovascular survival cohort study. We use a realistic model to synthesize data from the real-world covariates under varying conditions, *i.e*., different event rates, sample size, non-linearity, *etc*. More specifically, we use the CoxPH-Weibull model (Bender, Augustin, and Blettner 2005) to simulate the survival times of patients $T={\left\{\frac{-\text{log }U}{\lambda \text{ exp}(g(x))}\right\}}^{1/\nu }$, where *g*(*x*) is either a linear function or a randomly initialized neural net. Our goal is to predict whether the subject will decease within a pre-specified time frame, *i.e*., *T* < *t*_0_. Via adjusting the cut-off threshold *t*_0_, we can simulate different event rates. A detailed description of the simulation strategy is in the SM.

We experiment with different combinations of advanced VI techniques, as summarized in Table 1. Limited by space, we report results at 1% event rate with *g*(·) set to a randomly initialized neural network under various sample sizes. Additional results on linear models and other synthetic datasets are consistent and can be found in the SM. IAF and GPD only variants perform poorly, even compared to the vanilla VAE solution. This is possibly due to the fact that priors are mismatched. Explicitly matching to the prior via Fenchel mini-max learning technique improves performance. However, without using an encoder with a tractable likelihood, the model cannot directly leverage knowledge from the GPD prior likelihood. Stacked together (mixed GPD+IAF+Fenchel), our full proposal of VIE consistently outperforms its variants, approaching oracle performance in the large sample regime.

### Real-World Datasets

To extensively evaluate real-world performance, we consider a wide range of real-world datasets, briefly summarized below: (*i*) COVID: A dataset of patients admitted to the DUHS with positive COVID-19 testing, to predict death or use of a ventilator. (*ii*) InP (O’Brien et al. 2020): An in-patient data from DUHS, to predict the risk of death or ICR transfers. (*iii*) SEER (Ries et al. 2007): A public dataset studying cancer survival among adults curated by the U.S. Surveillance, Epidemiology, and End Results (SEER) Program, here we use a 10-year follow-up breast cancer subcohort. (*iv*) SLEEP (Quan et al. 1997): The Sleep Heart Health Study (SHHS) is a prospective cohort study about sleeping disorder and cardiovascular diseases. Summary statistics of these four real-world datasets are given in Table 3. Note that InP, SEER and SLEEP are all survival datasets, among which SEER and SLEEP include censored subjects. We follow the data pre-processing steps in (Xiu et al. 2020). To create outcome labels, we set a cut-off time to define an event of interest the same as in the ablation study, and exclude subjects censored before the cut-off time. The excluded samples only account for less than 0.2% of the whole population, and therefore it is expected to have a very limited impact on our results. Datasets have been randomly split into training, validation, and testing datasets with ratio 6:2:2. See the SM for details on data pre-processing.

Table 2 compares VIE to its counterparts, where the numbers are averaged over the bootstrap samples. We see the proposed VIE yields the best performance in almost all cases, and the lead is more significant with low event rates. Note that the one-class classification based DeepSVDD performs poorly, which implies treating rare events as outliers are inappropriate in the scenarios considered here. Reweighting and resampling based methods (IW, Focal) are less stable compared to those simple baselines (LASSO, MLP). The theoretically optimal LDAM works well in general, second only to VIE in most settings. To further demonstrate the stability of our method, we visualize the bootstrapped evaluation scores for the COVID dataset in Figure 3, and defer the additional cross-validation results to the SM. We see that VIE leads consistently.

**Figure 2:**
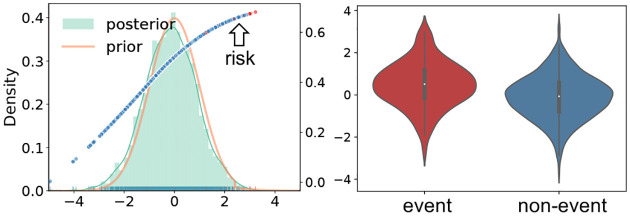
First latent dimension from the InP dataset (1% event rate). Left: Learned prior and posterior distribution, and monotonic predicted risks (right axis). Right: The latent representation values distribution grouped by event type.

**Figure 3:**
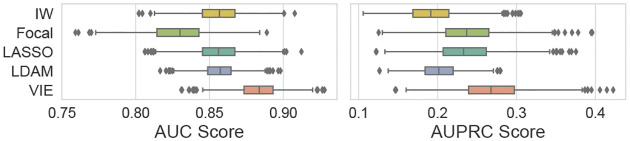
Bootstrapped AUC (left) and AUPRC (right) distributions for the COVID mortality data (2.6% event rate).

We also verify empirically that the estimated GPD shape parameters *ξ*_GPD_ are mostly positive (see the SM), indicating heavier than Gaussian tails as we have hypothesized. In Figure 2, we visualize one such latent dimension from the InP dataset, along with the associated risk learned by AMNN. In this example, the tail part is heavier than Gaussian and is associated with elevated risk. See our SM for examples where the extended tail contributes to prohibit the event.

## CONCLUSIONS

Motivated by the challenges of rare-event prediction in clinical settings, we presented Variational Inference with Extremals (VIE), a novel extreme representation learning-based variational solution to the problem. In this model we leveraged GPD to learn the extreme distributions with few samples and applied additive monotonic neural networks to disentangle the latent dimensions’ effects on the outcome. VIE featured better generalization and interpretability, as evidenced by a strong performance on real and synthetic datasets. In future work, we will extend this framework to the context of causal inference to quantify treatment effects in the label imbalanced setting (Lu et al. 2020).

## Supplementary Material

1

## Figures and Tables

**Table 1:** Ablation study of VIE with different combinations of architectures on realistic synthetic datasets with 1% event rate. The oracle model has used the ground-truth model parameters to predict.

					Average AUC (standard deviation)			Average AUPRC (standard deviation)		
	Prior	Encoder	Decoder	Prior Match	n=5k	n=10k	n=20k	n=5k	n=10k	n=20k
VAE			MLP	True	0.552 (0.092)	0.682 (0.030)	0.674 (0.020)	0.026 (0.010)	0.053 (0.010)	0.061 (0.017)
VAE-GPD	mixed GPD	Gaussian	AMNN	False	0.569 (0.062)	0.599 (0.010)	0.653 (0.027)	0.021 (0.003)	0.027 (0.005)	0.035 (0.013)
IAF-GPD	mixed GPD	IAF	AMNN	False	0.511(0.021)	0.551 (0.018)	0.665 (0.029)	0.017(0.002)	0.019 (0.002)	0.025 (0.008)
Fenchel-GPD	mixed GPD	Implicit	AMNN	True	0.623 (0.036)	0.668 (0.044)	0.694 (0.021)	0.037 (0.010)	0.048 (0.013)	0.062 (0.026)
VIE	mixed GPD	IAF	AMNN	True	**0.684** (0.031)	**0.697** (0.036)	**0.701** (0.017)	**0.050** (0.009)	**0.061** (0.025)	**0.079** (0.025)
Oracle (with 90% confidence interval)					0.704 [0.662, 0.751]			0.092 [0.058, 0.141]		

**Table 2:** Average AUC and AUPRC from real-world datasets.

	average AUC									average AUPRC								
	COVID		InP				SEER		SLEEP	COVID		InP				SEER		SLEEP
Event category	Mortality	Combined	12h	24h	48h	168h	3mo	llmo	600d	Mortality	Combined	12h	24h	48h	168h	3mo	llmo	600d
LASSO	0.856	0.853	0.822	0.789	0.767	0.760	0.888	0.845	0.720	0.235	**0.542**	0.092	0.131	0.159	0.216	0.140	0.309	0.164
MLP	0.862	0.854	0.824	0.806	0.762	0.768	0.885	0.856	0.730	0.225	0.531	0.093	0.141	0.159	0.221	0.169	0.322	0.182
DeepSVDD	NA	NA	0.633	0.608	0.605	0.551	0.592	0.572	0.644	NA	NA	0.020	0.030	0.044	0.063	0.026	0.068	0.118
IW	0.856	0.860	0.776	0.748	0.726	0.728	0.798	0.832	0.642	0.193	0.511	0.073	0.086	0.105	0.165	0.123	0.274	0.120
Focal	0.829	0.854	0.750	0.779	0.741	0.705	0.868	0.835	0.633	0.238	0.484	0.044	0.112	0.120	0.149	0.141	0.263	0.101
LDAM	0.857	0.843	0.819	0.805	0.785	0.774	0.893	0.861	0.755	0.202	0.535	0.086	0.130	0.148	0.197	0.177	0.332	0.179
VIE	**0.883**	**0.867**	**0.840**	**0.818**	**0.793**	**0.780**	**0.895**	**0.862**	**0.778**	**0.268**	0.535	**0.100**	**0.150**	**0.179**	**0.240**	**0.189**	**0.345**	**0.196**

**Table 3:** Summary statistics for real-world datasets.

	COVID	InP	SEER	SLEEP
sample size	25,315	67,655	68,082	5026
dimension	1268(668)	73(39)	789(771)	206(162)
event rate (%)	2.6%, 8%	1 ~ 5%	1 ~ 5%	5%

## Footnotes

1 SM can be found at https://arxiv.org/abs/2009.08541

2 We have removed the constant term for notational clarity
