# Supplementary material for: Variational Disentanglement for Rare Event Modeling
Source: ArXiv. 2020 Sep 17:arXiv:2009.08541v5. Preprint. [Version 5] (PMC7523120)
Supplement: 1 [file NIHPP2009.08541V5-supplement-1.pdf]

# Supplementary Material to "Variational Disentanglement for Rare Event Modeling"

## Contents

When the prevalence of an event is extremely low, but the event itself has substantial importance, the methods to identify such targets are called rare event modeling. Accurate and robust modeling of rare events is significant in many fields, such as identifying patients in high-risk and hopefully to prevent adverse outcomes from happening based on early intervention.

The scarcity of rare cases can cause extreme imbalanced among the dataset. Therefore, rare event modeling is challenging for most standard statistical approaches. As we discussed in the main text, careful statistical adjustments and new methodologies are required to approach such imbalance. Otherwise, the classifiers would be driven to the majority side and give misleading results. Also, the lack of representation in the minority class may cause unadjusted models to wrongly capturing spurious features that cannot generalize well to other observations. The apex of the risk curve or the mass of risk density usually overlays with the tail of the feature representation distribution, as illustrated in Figure S1, traditional statistical methods (such as Gaussian based approaches) often ill perform at the tail end, which can lead to lack-of-fit and poor generalization ability.

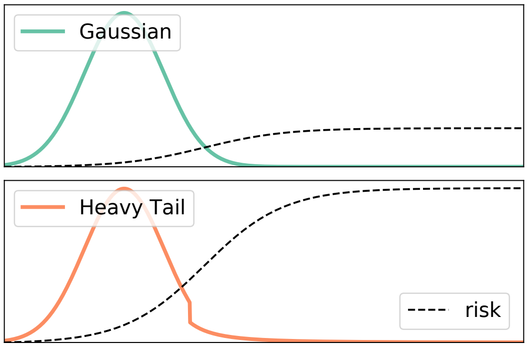


Figure S1: Feature representation mismatch at the tail parts. The heavy-tailed distribution can exploit extreme behavior in the latent space.

We approach a solution to such challenges with a variational representation learning scheme that models disentangled extreme representations. Further, we design a robust, powerful prediction arm that combines the merits of a generalized additive model and isotonic neural net.

## A. Derivation of Mixed GPD tail distribution

An important theory in *Extreme value theory* (EVT) shows that under some mild conditions, the conditional cumulative distribution of *exceedance* over a threshold $u$ follows Generalized Pareto Distribution, GPD$(u, \xi, \sigma)$ (McFadden 1978), which has the cumulative distribution function (CDF) as:

$$G_{\xi,\sigma,u}(x) = \begin{cases} 1 - [1 + \xi(x-u)/\sigma]^{-\frac{1}{\xi}}, & \text{if } \xi \neq 0 \\ 1 - \exp[-(x-u)/\sigma], & \text{if } \xi = 0 \end{cases}$$

where $\sigma$ is a positive scale parameter. According the shape parameter $\xi$, $x$ could have different support. When $\xi < 0$, the exceedance $x$ has bounded support $0 \leq x \leq u - \sigma/\xi$, otherwise $x$ is bounded by 0 on the left. $u$ is the location parameter. The corresponding PDF is:

$$g_{\xi,\sigma,u}(x) = \begin{cases} \sigma^{-1}[1 + \xi(x-u)/\sigma]^{-\frac{1}{\xi}-1}, & \text{if } \xi \neq 0 \\ \sigma^{-1}\exp[-(x-u)/\sigma], & \text{if } \xi = 0 \end{cases}$$

Thus the log-likelihood function is:

$$\log \text{likelihood}(x; \xi, \sigma, u) = \begin{cases} -\log\sigma - (\frac{1}{\xi}+1)\log[1 + \xi(x-u)/\sigma], & \text{if } \xi \neq 0 \\ -\log\sigma - (x-u)/\sigma, & \text{if } \xi = 0 \end{cases}$$

To enable modeling of the extreme representations, we adopt the Generalized Pareto Distribution as the tail part of our new variational prior, and the regular bulk representations $z \leq u$ with a standard Gaussian distribution. Then mixed extreme tail distribution has the form (McNeil 1997),

$$F(z) = P(Z \leq z) = P(Z \leq u) + (1 - P(Z \leq u))F_u(z-u)$$

When $z > u$, the tail estimator is,

$$\hat{F}(z) = (1 - F_n(u))G_{u,\xi,\sigma,u}(z) + F_n(u)$$

to approximate $F(z)$. Now we show that $\hat{F}(z)$ also has a GPD distribution with same $\xi$ and the following scale and location parameters,

$$\begin{cases} \tilde{\sigma} = \sigma(1 - F_n(u))^{\xi}, \tilde{u} = u - \tilde{\sigma}((1 - F_n(u))^{-\xi} - 1)/\xi, & \text{if } \xi \neq 0 \\ \tilde{\sigma} = \sigma, \tilde{u} = u + \tilde{\sigma} \log(1 - F_n(u)), & \text{if } \xi = 0 \end{cases}$$

When $\xi = 0$,

$$\begin{aligned} \hat{F}(z) &= (1 - F_n(u))(1 - \exp(-(x-u)/\sigma)) + F_n(u) \\ &= 1 - (1 - F_n(u)) \exp(-(x-u)/\sigma) \\ &= 1 - \exp(\log(1 - F_n(u))) \exp(-(x-u)/\sigma) \\ &= 1 - \exp(-\frac{1}{\sigma}(x - u - \sigma \log(1 - F_n(u)))) \\ &= 1 - \exp(-\frac{1}{\tilde{\sigma}}(x - \tilde{u})) \end{aligned}$$

When $\xi \neq 0$,

$$\begin{aligned} \hat{F}(z) &= (1 - F_n(u))(1 - (1 + \xi(x-u)/\sigma)^{-\frac{1}{\xi}}) + F_n(u) \\ &= 1 - (1 - F_n(u))(1 + \xi(x-u)/\sigma)^{-\frac{1}{\xi}} \\ &= 1 - [(1 - F_n(u))^{-\xi}(1 + \xi(x-u)/\sigma)]^{-\frac{1}{\xi}} \\ &= (1 - F_n(u))^{-\xi} + (1 - F_n(u))^{-\xi} \cdot \xi(x-u)/\sigma \\ &= \frac{1}{(1 - F_n(u))^{\xi}} + \frac{\xi(x-u)}{\sigma(1 - F_n(u))^{\xi}} \\ &= \frac{\sigma}{\tilde{\sigma}} + \frac{\xi(x-u)}{\tilde{\sigma}} \\ &= 1 + \frac{\sigma - \tilde{\sigma} + \xi(x - \tilde{u})}{\tilde{\sigma}} \\ &= 1 + \frac{\xi(x - \tilde{u} + \xi^{-1}\sigma - \xi^{-1}\tilde{\sigma})}{\tilde{\sigma}} \end{aligned}$$

Therefore, $\tilde{u} = \tilde{u} - \xi^{-1}\sigma - \xi^{-1}\tilde{\sigma}$.

## B. Implementation Details

Our main algorithm was written in PyTorch (version 1.3.1) (Paszke et al. 2017). The experiments were conducted on an Intel(R) Xeon(R) and Tesla P100-PCIE-16GB GPU (except for the `COVID` dataset). The `COVID` dataset were stored and analyzed on a protected virtual network space with Inter(R) Xeon(R) Gold 6152 CPU 2.10GHz 2 Core(s).

**Model Structure.** In VIE, we end up optimizing the following objective,

$$\max_{\theta,\phi} \min_{\nu} \{\mathbb{E}_{x,y\sim\mathcal{D}}[\Psi_\beta(x,y;p_\theta,q_\phi) - \lambda\Gamma(p_\theta,q_\phi,\nu)]\}, \tag{11}$$

where

$$\begin{aligned} \Psi_\beta(x,y;p_\theta,q_\phi) = \mathbb{E}_{Z\sim q_\phi(z|x)}&[\log p_\theta(y|Z)] \\ - \quad &\beta \mathrm{KL}[q_\phi(z|x)||p(z)], \end{aligned}$$

Note that the GPD parameters $(\xi, \sigma)$ are absorbed in $\phi$, and hyperparameter $u$ is used in the GPD prior $p(z)$. $u$ is set to be $F_z^{-1}(0.99)$ in all experiments. When the event rate is $\geq 1\%$, we set $\lambda, \beta = (1 \times 10^{-3}, 1 \times 10^{-5})$, otherwise we shrink the parameters to $\lambda, \beta = (1 \times 10^{-4}, 1 \times 10^{-6})$.

More concretely, the constituting parts $p_\theta(y|z)$, $p_\theta(z|x)$, $q_\phi(z|x)$ and $\nu(z)$ are specified as follows

$$\begin{aligned} p_\theta(y|z) &\leftarrow \Phi(H(z;\theta)) \text{ Log-Log link } (13), \\ H(z;\theta) &\leftarrow \text{ Additive Monotone Neural Net } (14) \text{ with} \\ p(z) &\leftarrow \text{Mixed GPD } (u, \xi_p, \sigma_p) \ (6), p = 4 \\ q_\phi(z|x) &\leftarrow \text{Inverse Autoregressive Flow } (8), \text{nstep} = 5 \\ \nu(z) &\leftarrow \text{Standard neural network.} \end{aligned} \tag{12}$$

Pseudo-code for VIE is presented in Algorithm 2. In all experiments, AMNN, IAF, $\nu(z)$ are specified in terms of two-layer MLPs of 32 hidden units with Rectified Linear Unit (ReLU) activation functions. The initial encoder `Init-Encoder` is

**Algorithm 2:** Variational Inference with Extremals.

**Data:** $\mathcal{D} = (x, y)$. $x$: inputs, $y$: labels
**Networks and parameters:** $\texttt{Init-Encoder}(x, \epsilon; \phi)$: Initial encoder network; $\texttt{IAF}(z; \phi)$: recursive autoregressive neural network; $\nu(z; \omega)$: critic neural network;
$\texttt{AMNN}(z; \theta)$: additive monotonic neural net;
prior: $p_\psi(z) = \texttt{MixedGPD}(z;\psi,u)$, $\psi=\{\xi_{\text{GPD}},\sigma_{\text{GPD}}\}$
**Initialize:** Init-Encoder, IAF, $\nu$, AMNN, $\psi$
**for** *iteration* $k \in \{1, \ldots, K\}$ **do**
  Sample $\{(x_i, y_i)\}_{i=1}^m$ from $\mathcal{D}$, $\{\epsilon_i\}_{i=1}^m$ from $p(\epsilon)$
  $[\mu_0, \sigma_0] = \texttt{Init-Encoder}(x, \epsilon; \phi)$
  Sample $z_{\text{pr}}$ from $p_\psi(z)$, $\boldsymbol{z}_0$ from $\mathcal{N}(\mu_0, \Sigma_0)$
  Compute $l_{\text{post}} := \log q_\phi(z_0|x)$
  **for** *step* $t \in \{1, \ldots, T\}$ **do**
    $[\mu_t, \sigma_t] = \texttt{IAF}(z_{t-1}; \phi)$, $z_t = \mu_t + \sigma_t \odot z_{t-1}$
    $l_{\text{post}} = l_{\text{post}} - \sum(\log \sigma_t)$
  **end**
  $\log p_\theta(y|z_T) = \ell_{\text{CLL}}(y, \text{AMNN}(z_T; \theta))$
  **Descend** $\omega$ by $\nabla_\omega \frac{1}{m} \sum [\nu_\omega(z_{\text{pr}}) - \log \nu_\omega(z_T)]$
  **Ascend** $\Omega = \{\phi, \psi, \theta\}$ by
  $\nabla_\Omega \frac{1}{m} \sum [\log p_\theta(y|z_T) - \log \nu_\omega(z_T) - \text{KL}]$, where $\text{KL} = l_{\text{post}} - \log p_\psi(z_T)$
**end**

specified as a three-layers MLPs of 32 hidden units. We set the minibatch size to $m = 200$. The critic network $\nu(x)$ uses the RMSprop optimizer with learning rate $1 \times 10^{-3}$, other parts of the algorithm used Adam optimizer with learning rate $1 \times 10^{-4}$. To avoid over-fitting, we set the dimension of latent space as 4 in all experiments.

Note we have used the *Complementary Log-Log* (CLL) link function for $p_\theta(y|z)$ in (13),

$$\Phi(a) = 1 - \exp(-\exp(a)), \tag{13}$$

for the outcome model as opposed to the standard *Logistic* link $1/(1 + \exp(-a))$. The CLL link is more sensitive at the tail end, so it is more frequently used in statistical models dealing with vanishing probabilities (Aranda-Ordaz 1981).

To avoid collapsing to suboptimal local minimums, we train the encoder arm more frequently to compensate for the detrimental posterior lagging phenomenon (He et al. 2019). Our pseudo-code for VIE is summarized in Algorithm 1.

**Numerical Integration.** Following (5), we divide region $[l, z_j]$ evenly into $M$ bins of width $d_j = \frac{z_j - l}{M}$, with $z_{j,M} = z_j$. For the $M$ bins, we select a random point $z_{j,k}^r$ in each bin. The integral approximation on support $[z_{j,k}, z_{j,k+1}]$ is the rectangular area $h_j(z_{j,k}^{(r)}) * d_j$. As a result, the integral $\int_0^{z_j} h(s; \theta) ds$ is approximated with $\sum_k^{M-1} h_j(z_{j,k}^{(r)}) d_j$. With this approximation (5) can be written as:

$$H(z; \theta) = \sum_j^p \alpha_j d_j \sum_k^M h_j(z_{j,k}^{(r)}) + \gamma. \tag{14}$$

We set $M = 100$ and $l = -5$ in all the experiments.

**Discussions on Evaluation Metrics.** In the main text, we reported AUC and AUPRC instead of single evaluation metrics, *e.g.*, overall accuracy or error rate. Standard statistical metrics like Brier Scores (BS) and Binary classification entropy (BCE) could be deceptive when the event rate is low, *e.g.*, $\leq 10\%$ (Schmid and Griffith 2014). We will add BCE loss and the positive case BCE loss in the following sections in the simulation study for reference. Some poorly performed models can have relatively low BCE scores. In this case, the ground truth (Oracle) is the best reference we have.

## C. Ablation Study

We examine model performance on two simulation strategies. The first one is the semi-synthetic dataset, which exploits the real-world covariates structures. The second one is a synthetic dataset based on our extreme representation assumptions.

**Semi-synthetic Datasets** We synthesize a semi-synthetic dataset based on the Framingham study (Mitchell et al. 2010), a long-term cardiovascular survival cohort study. After quality control, $40,046$ subjects with nine covariates (four continuous and five categorical) are included.

We use a realistic model to synthesize data from the real-world covariates under varying conditions, *i.e.*, different event rates, sample size, nonlinearity, *etc.* More specifically, we use the coxPH-Weibull model (Bender, Augustin, and Blettner 2005) to

simulate the survival time of patients $T = \{\frac{-\log U}{\lambda \exp(g(x))}\}^{1/\nu}$, where $g(x)$ is either a linear function or a randomly initialized neural net. Our goal is to predict whether the subject will decease within a pre-specified time frame, *i.e.*, $T < t_0$. Via adjusting the cut-off threshold $t_0$, we can simulate different event rates. The details are shown in Algorithm 3.

**Algorithm 3:** Semi-synthetic Data

```
Extract covariates from Framingham Dataset ;
Set ν, λ (the parameters of cox-Weibull distribution);
Set time cut-bound t_0;
Decide g(x) : R^q → R form;
for i ∈ {1, ..., n} do
    Sample u_i from Unif(0, 1);
    Compute t_i = {−log u_i / (λ exp(g(x_i)))}^{1/ν};
    Compute y_i = 1[t_i < t_0];
    d_i = (y_i, x_i);
end
return D = {d_i; i = 1 ... n}
```

In our experiments, the performances when $g(\cdot)$ set as a randomized neural network or a linear function do not differ very much. For simplicity, we will present the results under the neural network settings. Apart from the results at $1\%$ event rate given in the main text, we will show the results at $0.5\%$ and $5\%$ event rates here. The oracle results are calculated with plugging in the true $g(x_i)$ in Algorithm 3, and the randomness is from the generating scheme of the survival time $t$.

**Additional Results for semi-synthetic datasets** In $1\%$ event rate case presented in the main text, the AUC and AUPRC distributions are summarized in Figure S2, which corresponds to the average and standard deviation values presented in Table 1. We further examine the cases with $0.5\%$ and $5\%$ event rates to evaluate our method's robustness. Results are summarized in

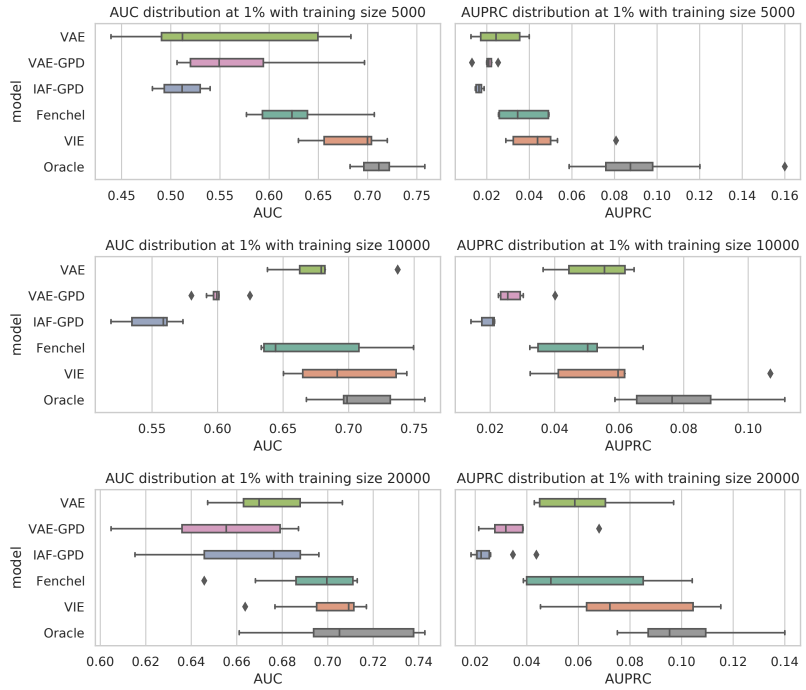


Figure S2: Box plot of 10 independent $1\%$ event rate semi-synthetic analysis.

Table S1 and Table S2 respectively. Apart from the threshold-free metrics AUC and AUPRC, we also presented Binary Cross-Entropy loss (BCE) and the BCE loss associated with true events (positive case losses). Note that for an imbalanced dataset, BCE loss can be misleading. In the model, VAE-GPD, which is poorly-behaved in AUC and AUPRC, can have relatively low BCE loss since the majority group overwhelms the minority (*more important*) group. We can refer to the BCE loss and positive

case loss in the oracle results for reference. VIE performs consistently close to the oracle results, especially with low event rate and small training sample size, and Fenchel-GPD is in the second place.

Table S1: Ablation study of VIE with $0.5\%$ event rate in semi-synthetic settings.

| | Average AUC (std) ↑ | | | Average AUPRC (std)↑ | | | Average BCE Loss (std) ↓ | | | Average Positive Case Loss (std) ↓ | | |
|---|---|---|---|---|---|---|---|---|---|---|---|---|
| | n=5k | n=10k | n=20k | n=5k | n=10k | n=20k | n=5k | n=10k | n=20k | n=5k | n=10k | n=20k |
| VAE | 0.494 (0.111) | 0.623 (0.102) | 0.697 (0.061) | 0.007 (0.005) | 0.017 (0.010) | 0.020 (0.007) | 0.498 (0.309) | **0.035** (0.005) | **0.031** (0.004) | **0.010** (0.010) | 0.027 (0.005) | 0.026 (0.005) |
| VAE-GPD | 0.560 (0.045) | 0.602 (0.044) | 0.635 (0.045) | 0.008 (0.002) | 0.013 (0.010) | 0.016 (0.003) | 5.250 (2.096) | 0.768 (0.258) | 0.152 (0.222) | 0.000 (0.000) | **0.004** (0.001) | **0.018** (0.008) |
| IAF-GPD | 0.631 (0.039) | 0.555 (0.042) | 0.533 (0.061) | 0.011 (0.007) | 0.008 (0.002) | 0.017 (0.013) | **0.032** (0.003) | 0.038 (0.008) | 0.043 (0.014) | 0.027 (0.003) | 0.024 (0.001) | 0.027 (0.007) |
| Fenchel-GPD | 0.615 (0.059) | 0.652 (0.055) | 0.667 (0.024) | 0.022 (0.016) | 0.021 (0.012) | **0.025** (0.008) | 0.034 (0.004) | 0.037 (0.006) | 0.033 (0.002) | 0.028 (0.005) | 0.032 (0.006) | 0.027 (0.002) |
| VIE | **0.654** (0.074) | **0.692** (0.076) | **0.693** (0.036) | **0.022** (0.010) | **0.027** (0.015) | 0.024 (0.009) | 0.041 (0.018) | 0.036 (0.003) | 0.032 (0.003) | 0.026 (0.005) | 0.030 (0.005) | 0.026 (0.003) |
| Oracle | 0.688 (0.618, 0.769) | | | 0.043 (0.023, 0.071) | | | 0.034 (0.028, 0.040) | | | 0.029 (0.023, 0.035) | | |

Table S2: Ablation study of VIE with $5\%$ event rate in semi-synthetic settings.

| | Average AUC (std) ↑ | | | Average AUPRC (std)↑ | | | Average BCE Loss (std) ↓ | | | Average Positive Case Loss (std) ↓ | | |
|---|---|---|---|---|---|---|---|---|---|---|---|---|
| | n=5k | n=10k | n=20k | n=5k | n=10k | n=20k | n=5k | n=10k | n=20k | n=5k | n=10k | n=20k |
| VAE | 0.594 (0.118) | 0.666 (0.021) | 0.693 (0.011) | 0.113 (0.049) | 0.144 (0.017) | 0.179 (0.018) | 0.308 (0.166) | 0.198 (0.004) | 0.198 (0.009) | 0.111 (0.046) | 0.147 (0.003) | 0.149 (0.008) |
| VAE-GPD | 0.583 (0.027) | 0.607 (0.014) | 0.663 (0.009) | 0.075 (0.008) | 0.087 (0.013) | 0.137 (0.024) | 2.106 (1.553) | 0.581 (0.056) | 0.195 (0.011) | **0.017** (0.015) | **0.043** (0.004) | 0.143 (0.013) |
| IAF-GPD | 0.664 (0.017) | 0.554 (0.033) | 0.503 (0.020) | 0.113 (0.022) | 0.063 (0.007) | 0.057 (0.003) | 0.194 (0.008) | 0.208 (0.004) | 0.214 (0.009) | 0.144 (0.009) | 0.157 (0.004) | 0.160 (0.008) |
| Fenchel-GPD | 0.666 (0.014) | 0.687 (0.016) | 0.681 (0.008) | **0.145** (0.011) | **0.184 (0.022)** | 0.166 (0.013) | 0.196 (0.008) | **0.189** (0.007) | 0.190 (0.011) | 0.148 (0.008) | 0.141 (0.007) | 0.141 (0.012) |
| VIE | **0.679** (0.018) | **0.693** (0.027) | **0.693** (0.015) | 0.142 (0.018) | 0.172 (0.032) | **0.193**(0.013) | **0.188** (0.011) | 0.193 (0.005) | **0.190** (0.006) | 0.139 (0.013) | 0.145 (0.006) | **0.139** (0.008) |
| Oracle | 0.694 (0.670, 0.717) | | | 0.197 (0.179, 0.218) | | | 0.185 (0.179, 0.198) | | | 0.137 (0.130, 0.149) | | |

**Long-tailed Synthetic Datasets** We design the long-tailed synthetic datasets based on our proposed method, where the latent variable $z$ enjoys a long-tailed distribution. The pseudo-code for this simulation strategy is shown in Algorithm 4, where $t_0$ is a pre-specified time-cut, and $H(\cdot)$ is a randomized monotone neural network to create a monotone mapping from $z$ to the risk.

**Algorithm 4:** Generation of long-tailed data.

Set sample size $n$, latent space dimension $p$, number of covariates $q$ ;
Set $\mu_p, \Sigma_p, \xi_p, \sigma_p$ (the parameters of a long-tailed distribution);
Set $\nu$, $\lambda$ (the parameters of cox-Weibull distribution);
Set time cut-bound $t_0$;
Initialize $\psi$ (for MLP $g(\cdot;\psi) : \mathbb{R}^p \rightarrow \mathbb{R}^q$), $\theta$ (for AMNN $H(\cdot;\theta) : \mathbb{R}^p \rightarrow \mathbb{R}$);
**for** $i \in \{1, \ldots, n\}$ **do**
  Sample $z_i$ from mixed GPD $(\mu_p, \Sigma_p, \xi_p, \sigma_p)$;
  Compute $x_i = g(z_i; \psi)$;
  Sample $u_i$ from $\texttt{Unif}(0, 1)$;
  Compute $t_i = \{\frac{-\log u_i}{\lambda \exp(H(z_i;\theta))}\}^{1/\nu}$;
  Compute $y_i = I(t_i < t_0)$;
  $d_i = (y_i, x_i)$
**end**
;
**return** $D = \{d_i; i = 1 \ldots n\}$

Table S3 summarizes the findings with the long-tailed distributed latent space datasets, with $1\%$ event rate. VIE can achieve relatively high AUC and AUPRC even with a small training sample size, which suggests that the proposed method can recover the long-tailed behavior in the feature representation. Among the combinations of different VI techniques, the Fenchel duality mechanism facilitates the distribution matching the best among other inference techniques.

Table S3: Ablation study of VIE with $1\%$ event rate in longtailed-synthetic settings

| | Average AUC (std) ↑ | | | Average AUPRC (std)↑ | | | Average BCE Loss (std) ↓ | | | Average Positive Case Loss (std) ↓ | | |
|---|---|---|---|---|---|---|---|---|---|---|---|---|
| | n=5k | n=10k | n=20k | n=5k | n=10k | n=20k | n=5k | n=10k | n=20k | n=5k | n=10k | n=20k |
| VAE | 0.722 (0.140) | 0.741 (0.099) | 0.798 (0.034) | 0.128 (0.076) | 0.119 (0.064) | 0.177 (0.034) | 0.138 (0.213) | 0.121 (0.200) | 0.055 (0.004) | 0.029 (0.010) | 0.034 (0.009) | 0.039 (0.005) |
| VAE-GPD | 0.498 (0.039) | 0.450 (0.021) | 0.441 (0.055) | 0.013 (0.002) | 0.009 (0.000) | 0.009 (0.002) | 12.097 (5.582) | 22.445 (10.247) | 13.438 (7.159) | 0.000 (0.000) | 0.000 (0.000) | 0.001 (0.002) |
| IAF-GPD | 0.688 (0.021) | 0.632 (0.037) | 0.555 (0.039) | 0.097 (0.019) | 0.078 (0.027) | 0.046 (0.014) | 0.051 (0.005) | 0.055 (0.003) | 0.062 (0.007) | 0.040 (0.005) | 0.044 (0.004) | 0.051 (0.007) |
| Fenchel-GPD | 0.804 (0.028) | 0.807 (0.026) | 0.818 (0.020) | 0.174 (0.030) | 0.155 (0.052) | 0.166 (0.044) | 0.054 (0.007) | 0.051 (0.003) | **0.047** (0.004) | 0.041 (0.007) | 0.040 (0.004) | 0.037 (0.005) |
| VIE | **0.823** (0.024) | **0.810** (0.023) | **0.836** (0.026) | **0.175** (0.036) | **0.163** (0.044) | **0.202** (0.024) | **0.047** (0.005) | **0.050** (0.003) | 0.049 (0.005) | **0.037** (0.004) | **0.040** (0.003) | **0.037** (0.005) |
| Oracle | 0.829 (0.802, 0.868) | | | 0.188 (0.153, 0.243) | | | 0.049 (0.042, 0.055) | | | 0.039 (0.033, 0.045) | | |

In summary, we have tested the performance on various simulation settings (model assumptions, event rates, sample sizes, non-linearity, *etc.*) where VIE takes the lead in all cases. IAF- and GPD-only variants perform poorly, even not comparable to the vanilla VAE model. This is possibly due to the prior is not matched. Explicitly matching the prior via the Fenchel mini-max

scheme improves the performance, especially in the long-tailed representation datasets. Stacked together, our full proposal of VIE consistently outperforms its variants and always approaching the oracle performance in the large sample regime.

## D. Real-world datasets

We consider 5 real-world datasets, including 3 survival datasets in the study. Among those dataset, `COVID` and `InP` are from Duke University Health System (DUHS), which are not public at this time. `SEER`(Ries et al. 2007) and `SLEEP`(Quan et al. 1997) are two public survival datasets. Besides above clinical-based datasets, we further evaluate the model performance on `Fraud` dataset (Dal Pozzolo et al. 2017) in this supplementary material.

**Baseline Models.** In all experiments, LDAM, FOCAL, IW, DeepSVDD and MLP are specified in terms of three-layer MLPs of 32 hidden units with ReLU activation. When tuning parameters for LASSO, based on the notation of Pedregosa et al. (2011) function `sklearn.linear_model.Lasso`, we choose $\alpha$ from $[10^{-5}, 10^{-4}, 10^{-3}, 10^{-2}, 0.1, 0.2, 0.5, 0.8]$ referred to the best performance on the validation datasets. In Focal Loss, the parameter $\gamma$ is selected from the list $[0.1, 0.5, 1.0, 1.5, 2.0]$ based on the best performance on the validation datasets.

**COVID Dataset** The dataset includes inpatient encounters to DUHS as of January 1, 2020. Vitals, administered medications, lab results, comorbidities, etc. are used as predictors to identify the risk of inpatient death, ventilation, and ICU transfer as adverse outcomes. The raw data's detailed description for each group of covariates can be found in Table S4. The mortality rate in this dataset is $2.8\%$, ventilation $7.8\%$ and ICU transfer $18\%$. From rare event modeling purposes, apart from the mortality prediction, we set the group of patients who experienced either death or ventilation as the combined adverse outcome group, which has $8\%$ event rate.

Table S4: Raw `COVID` dataset covariates before pre-processing.

| Data Name | Data Type | Number Covariates |
|---|---|---|
| Demographics | Numerical | 1 (age) |
| Previous Encounters | Numerical | 2 |
| Prior Procedures w/n year | Categorical | 186 |
| Problem List w/n year | Categorical | 273 |
| Comorbidities w/n year | Categorical | 545 |
| Chief Complaint | Categorical | 100 |
| Lab Analytes Collected | Categorical | 44 |
| Lab Analytes Results | Numerical | 44 |
| Orders Placed | Categorical | 32 |
| Medications Administered | Categorical | 74 |
| Vitals Recorded | Numerical | 37 |

In Figure S3, we presented the comparison of VIE versus other baseline models with the two outcomes (combined and mortality). VIE shows strong performance under these metrics.

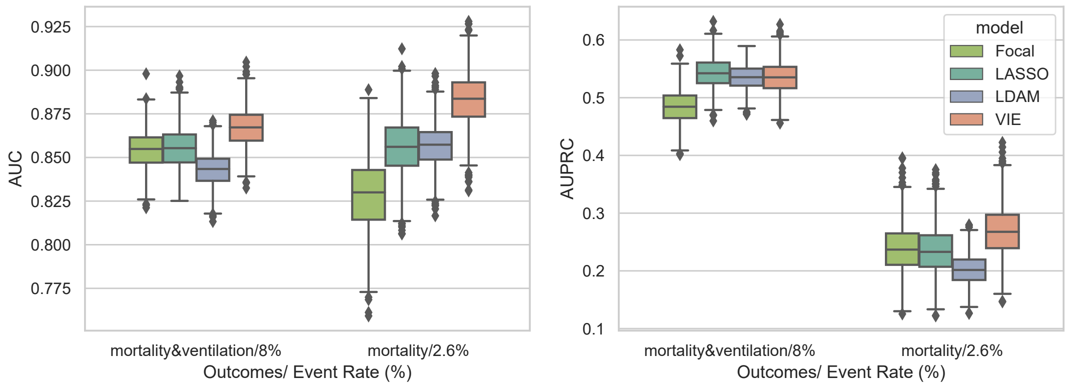


Figure S3: Bootstrapped AUC (left) and AUPRC (right) Distribution of COVID datset with different outcomes. Note that comparisons of AUPRC among event-rates groups are meaningless.

**Cross-validation results** To qualitatively show the superior performance of VIE, we examine the performances on a 5-fold cross validation of the `COVID` dataset for mortality prediction. VIE outperforms other baseline consistentlly on each fold.

Comparing to the performance of the second-best model LDAM with a paired t test, the p-value yields $0.093 < 0.1$, with effect size 0.98, which shows the performance gap is statistically significant at $\alpha = 0.1$.

Table S5: 5-Fold cross validation results for COVID-19 dataset

| | AUC | | | | | AUPRC | | | | |
|---|---|---|---|---|---|---|---|---|---|---|
| k-Fold | 1 | 2 | 3 | 4 | 5 | 1 | 2 | 3 | 4 | 5 |
| LASSO | 0.845 | 0.834 | 0.819 | 0.818 | 0.817 | 0.234 | 0.183 | 0.213 | 0.185 | 0.181 |
| VAE | 0.831 | 0.842 | 0.848 | 0.800 | 0.764 | 0.198 | 0.181 | 0.191 | 0.196 | 0.147 |
| MLP | 0.852 | 0.836 | 0.845 | 0.852 | 0.821 | 0.248 | 0.174 | 0.236 | 0.235 | 0.182 |
| Focal | 0.847 | 0.837 | 0.836 | 0.851 | 0.836 | 0.229 | 0.158 | 0.216 | 0.241 | 0.186 |
| LDAM | 0.842 | 0.848 | 0.843 | 0.839 | 0.839 | 0.240 | 0.197 | 0.234 | 0.209 | 0.182 |
| VIE | **0.860** | **0.849** | **0.851** | **0.865** | **0.840** | **0.263** | **0.210** | **0.238** | **0.256** | **0.201** |

**InP Dataset** The dataset is another inpatient data of 82,450 Duke University Health System (DUHS) collected between 2014-2016. We abstracted time-varying clinical data (*i.e.*, vital signs, laboratory tests, medications) and followed patients until the Intensive Care Unit (ICU) transfer or Death. We extracted their first encounter in the system (the admission) to generate this classification study to predict the risk of the occurrence of adverse outcomes (death or ICU transfer). The descriptions of raw data can be found in Table S6. With different sizes of the time windows, we generate four classification datasets with different event rates. As summarized in Figure S4, VIE takes a consistent lead in both AUC and AUPRC. The advantage enlarges when the event rates drop. Note that the trend of AUPRC when event rate shrinking is not meaningful(Boyd et al. 2012).

Table S6: Raw `InP` dataset covariates before pre-processing

| Data Name | Data Type | Number Covariates |
|---|---|---|
| Demographics | Numerical | 3 (age, sex, race) |
| Admission Information | Categorical | 2 (source and department) |
| Vitals Recorded | Numerical | 10 (Diastolic, Resp, SpO2, etc.) |
| Lab Analytes Collected | Categorical | 30 |
| Lab Analytes Results | Numerical | 30 |
| Lab Orders Placed | Categorical | 30 |

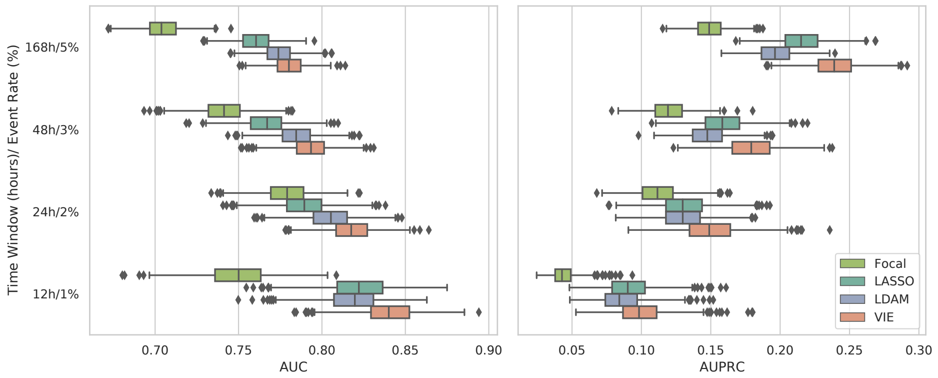


Figure S4: Bootstrapped AUC (left) and AUPRC (right) Distribution of InP datset with different event rate. Note that comparisons of AUPRC among event-rates groups are meaningless.

**SEER and SLEEP Datasets** `SEER` and `SLEEP` are two public survival datasets that contain censoring (*i.e.,* an event that is not reported during the follow-up period of a subject). To create a classification dataset from a survival dataset, we deleted patients censored before the time-cut. The proportion of subjects excluded for `SEER` is less than $0.1\%$, for `SLEEP` dataset is less than $0.2\%$, which should not affect the overall credibility of the analysis. We follow the pre-processing steps provided in Chapfuwa et al. (2020).

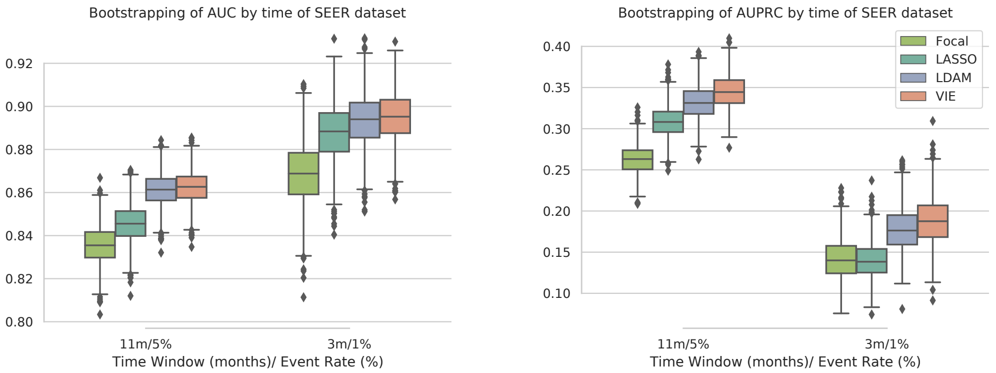


Figure S5: Bootstrapped AUC (left) and AUPRC (right) Distribution of SEER dataset with different event rate.

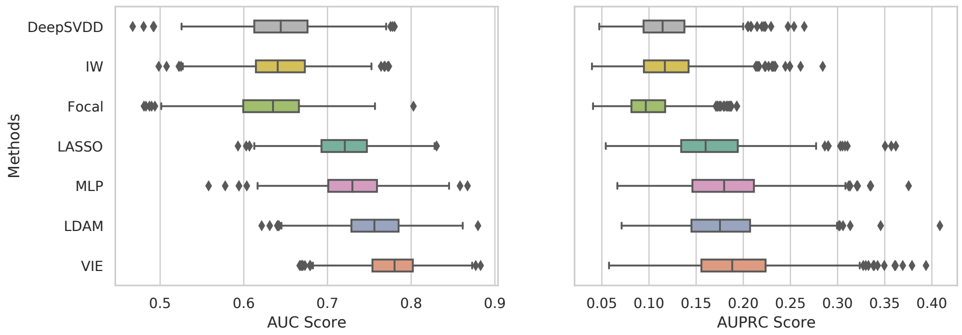


Figure S6: Bootstrapped AUC (left) and AUPRC (right) Distribution of SLEEP dataset with 5% event rate.

**Credit Card Fraud Detection** To evaluate the performances on non-clinical data, we examined the VIE model on fraud detection benchmark dataset (Dal Pozzolo et al. 2017), where fraudulent credit card transactions are coined as rare events ($\sim 0.2\%$). The dataset includes 284k records with 29 covariates. We split the original dataset into training, validation, and testing datasets with a 6:2:2 ratio to ensure fair and stable comparison. The hyperparameters are selected based on the best performance on the validation dataset. The average and standard deviation of the metrics are presented in Table S7. VIE outperforms other baselines and achieved an average of over $0.99$ AUC in the bootstrapped samples.

Table S7: Fraud transaction classification.

| | Lasso | MLP | DeepSVDD | IW | Focal | LDAM | VIE |
|---|---|---|---|---|---|---|---|
| AUC | 0.981 (0.006) | 0.984 (0.007) | 0.796 (0.019) | 0.777 (0.026) | 0.916 (0.020) | 0.987 (0.005) | **0.991** (0.003) |
| AUPRC | 0.79 (0.032) | 0.80 (0.030) | 0.01 (0.002) | 0.57 (0.039) | 0.79 (0.032) | 0.78 (0.037) | **0.80** (0.032) |

**Exploration of the feature representation** We visualize the marginal relationship between latent space dimensions and risk in the real-world dataset `InP` dataset ($1\%$ event rate), which are shown in Figure S7. The first dimension (top-left), the extremal behavior contributes significantly and positively to the event risk prediction. The other three dimensions serve as inhibitors to the event risk. Empirically, all the latent dimensions have a long-tailed distribution, with learned scale parameter $\xi > 0$.

We also embed the posterior space $z$ on a 2D plot with $t$-SNE, with probability contour lines, as shown in Figure S8. The events are concentrated to one end of the latent space.

## E. Generalized to Multiple-class classification

Our binary classification framework can be generalized to multiple-class problems easily. We will stick to the mixed-GPD distribution of the posterior $z$ with $p$ dimensions, and increase the number of monotone networks for each dimension of $z$. In the binary case, each dimension of $z$ corresponds to one monotone network, here we can set it to $k$ networks per-dimension. In total, we now have $p \times k$ monotone functions. Then we can apply an FC layer to the final output, with $m$ categories, as shown in Figure S9. In the learning object, we would replace the binary cross-entropy loss (BCE) with regular cross-entropy loss (CE) in the reconstruction term.

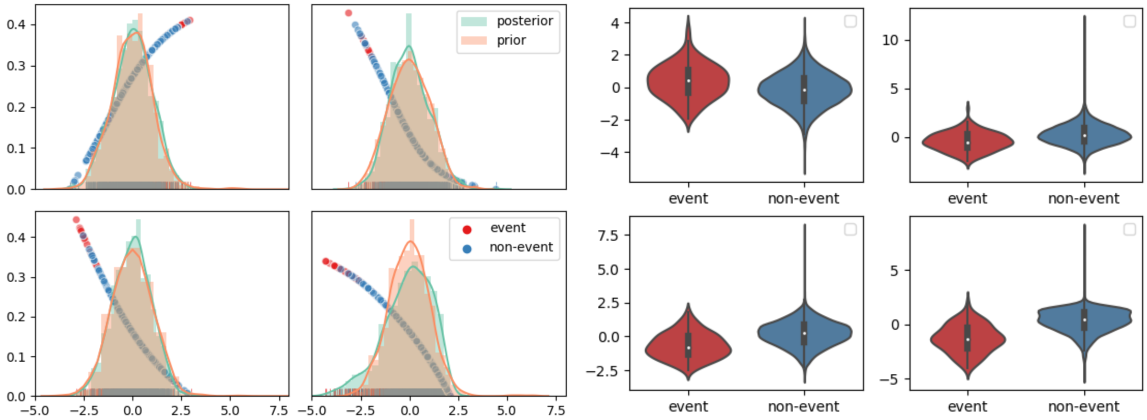


(a) Learned prior and posterior distribution and monotonic predicted risks

(b) The latent representation values distribution grouped by event type

Figure S7: Four latent dimensions from the InP dataset (1%) event rate, where the extreme distribution in the first dimension is the simulator to the events, the other three dimensions serve as inhibitors

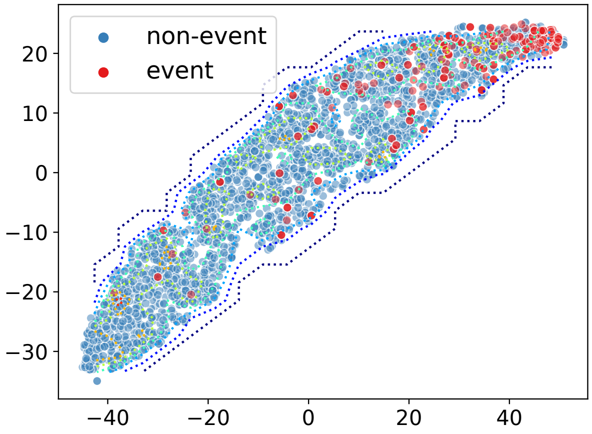


Figure S8: $t$-SNE plots with latent representation $z$.

$$
\begin{aligned}
p_\theta(y|z) &\leftarrow \Phi(H(z;\theta)) \text{ Soft-max },\\
H(z;\theta) &\leftarrow k \text{ Additive Monotone Neural Nets (14)}\\
p(z) &\leftarrow \text{Mixed GPD } (u, \xi_p, \sigma_p)\ (6),\\
q_\phi(z|x) &\leftarrow \text{Inverse Autoregressive Flow } (8),\\
\nu(z) &\leftarrow \text{Standard neural network.}
\end{aligned} \tag{15}
$$

We generate a toy dataset to illustrate VIE's performance on multiclassification problems. Based on Algorithm 3, instead of setting a binary time-cut, now we split the generated time $t$ with a sequence of time-cuts based on the percentiles $[5\%, 15\%, 30\%, 60\%]$ of $t$. In this way, we have a dataset with 5 categorical outcomes with event rates $[5\%, 10\%, 15\%, 30\%, 40\%]$, respectively. To evaluate the performance, except for the per-class accuracy, we use $F1$ score, which is a the harmonic mean of precision (True Positives) and recall (sensitivity), $\frac{2}{\text{recall}^{-1}+\text{precision}^{-1}}$, ranges from 0 to 1, where 1 indicating better performance. We use micro-averaged F1-score (micro-F1) to calculate the overall F1 scores for all classes,

Comparing to related methods: FOCAL and LDAM, the model VIE results on this toy example are comparable per class and better in terms of F1 score. FOCAL and LDAM are specified as 3-layer MLPs with 32 hidden units, and VIE uses the previous setting, except for $k = 3$.

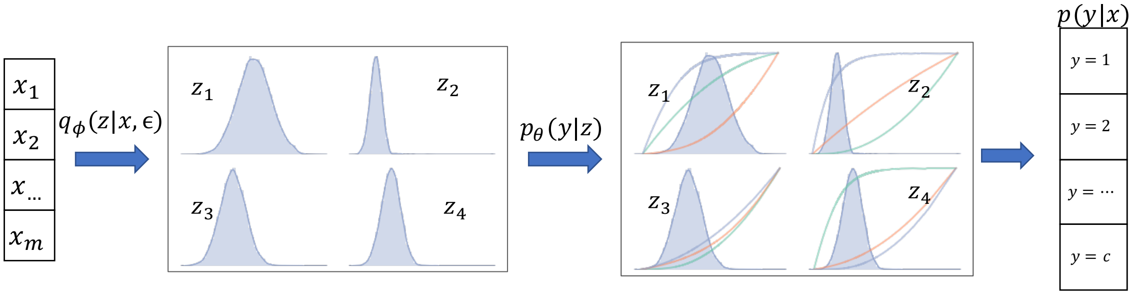


Figure S9: Illustration of multi-classification framework.

Table S8: Performance on few-shots learning dataset

| | class 1 | class 2 | class 3 | class 4 | class 5 | micro-F1 |
|---|---|---|---|---|---|---|
| event rates | 5% | 10% | 15% | 30% | 40% | |
| Focal | **0.503** | 0.202 | 0.095 | 0.139 | 0.089 | 0.1368 |
| LDAM | 0.012 | **0.321** | **0.228** | 0.165 | 0.594 | 0.3522 |
| VIE | 0.054 | 0.054 | 0.046 | **0.372** | **0.823** | **0.4521** |

## SM References

Chapfuwa, Paidamoyo, Chunyuan Li, Nikhil Mehta, Lawrence Carin and Ricardo Henao. 2020. Survival cluster analysis. In *Proceedings of the ACM Conference on Health, Inference, and Learning*. pp. 60–68.

Dal Pozzolo, Andrea, Giacomo Boracchi, Olivier Caelen, Cesare Alippi and Gianluca Bontempi. 2017. “Credit card fraud detection: a realistic modeling and a novel learning strategy.” *IEEE transactions on neural networks and learning systems* 29(8):3784–3797.

McFadden, Daniel. 1978. “Modeling the choice of residential location.” *Transportation Research Record* (673).

McNeil, Alexander J. 1997. “Estimating the tails of loss severity distributions using extreme value theory.” *ASTIN Bulletin: The Journal of the IAA* 27(1):117–137.

Paszke, Adam, Sam Gross, Soumith Chintala, Gregory Chanan, Edward Yang, Zachary DeVito, Zeming Lin, Alban Desmaison, Luca Antiga and Adam Lerer. 2017. “Automatic differentiation in PyTorch.”.

Pedregosa, F., G. Varoquaux, A. Gramfort, V. Michel, B. Thirion, O. Grisel, M. Blondel, P. Prettenhofer, R. Weiss, V. Dubourg, J. Vanderplas, A. Passos, D. Cournapeau, M. Brucher, M. Perrot and E. Duchesnay. 2011. “Scikit-learn: Machine Learning in Python.” *Journal of Machine Learning Research* 12:2825–2830.

Quan, Stuart F, Barbara V Howard, Conrad Iber, James P Kiley, F Javier Nieto, George T O’Connor, David M Rapoport, Susan Redline, John Robbins, Jonathan M Samet et al. 1997. “The sleep heart health study: design, rationale, and methods.” *Sleep* 20(12):1077–1085.

Ries, LA Gloeckler, JL Young, GE Keel, MP Eisner, YD Lin, MJ Horner et al. 2007. “SEER survival monograph: cancer survival among adults: US SEER program, 1988-2001, patient and tumor characteristics.” *National Cancer Institute, SEER Program, NIH Pub* (07-6215):193–202.

Schmid, Christopher H and John L Griffith. 2014. “Multivariate classification rules: calibration and discrimination.” *Wiley StatsRef: Statistics Reference Online* .
